# Supplementary material for: Chitosan Covalently Functionalized with Peptides Mapped on Vitronectin and BMP-2 for Bone Tissue Engineering
Source: Nanomaterials (Basel). 2021 Oct 21;11(11):2784. doi: 10.3390/nano11112784 (PMC8622029; doi:10.3390/nano11112784)
Supplement: Supplementary file 1 [file nanomaterials-11-02784-s001.zip › nanomaterials-1410116-supplementary.pdf]

Supplementary Materials

Chitosan Covalently Functionalized with Peptides Mapped on Vitronectin and BMP-2 for Bone Tissue Engineering

Paola Brun [1,†], Annj Zamuner [2,3,†], Leonardo Cassari [2], Gabriella D'Auria [4], Lucia Falcigno [4], Stefano Franchi [5], Giorgio Contini [5,6], Martina Marsotto [7], Chiara Battocchio [7], Giovanna Iucci [7] and Monica Dettin [2,3,*]

1 Department of Molecular Medicine, University of Padova, Via A. Gabelli 63, 35121 Padova, Italy; paola.brun.1@unipd.it
2 Department of Industrial Engineering, University of Padova, Via F. Marzolo 9, 35131 Padova, Italy; annj.zamuner@unipd.it (A.Z.); leonardo.cassari@phd.unipd.it (L.C.)
3 L.i.f.e.L.a.b. Program, Consorzio per la Ricerca Sanitaria (CORIS), Veneto Region, 35128 Padova, Italy
4 Department of Pharmacy, University Federico II of Naples, Via Domenico Montesano 49, 80131 Naples, Italy; gabriella.dauria@unina.it (G.D.); falcigno@unina.it (L.F.)
5 Istituto di Struttura Della Materia-CNR (ISM-CNR), Via Fosso del Cavaliere 100, 00133 Roma, Italy; stefano.franchi79@gmail.com (S.F.); giorgio.contini@ism.cnr.it (G.C.)
6 Department of Physics, University of Rome Tor Vergata, Via Della Ricerca Scientifica 1, 00133 Roma, Italy
7 Department of Science, Roma Tre University of Rome, Via Della Vasca Navale 79, Rome 00146, Italy; martina.marsotto@uniroma3.it (M.M.); giovanna.iucci@uniroma3.it (C.B.); giovanna.iucci@uniroma3.it (G.I.)
* Correspondence: monica.dettin@unipd.it; Tel. +39-0498-2755-53
† These authors contributed equally to this study.

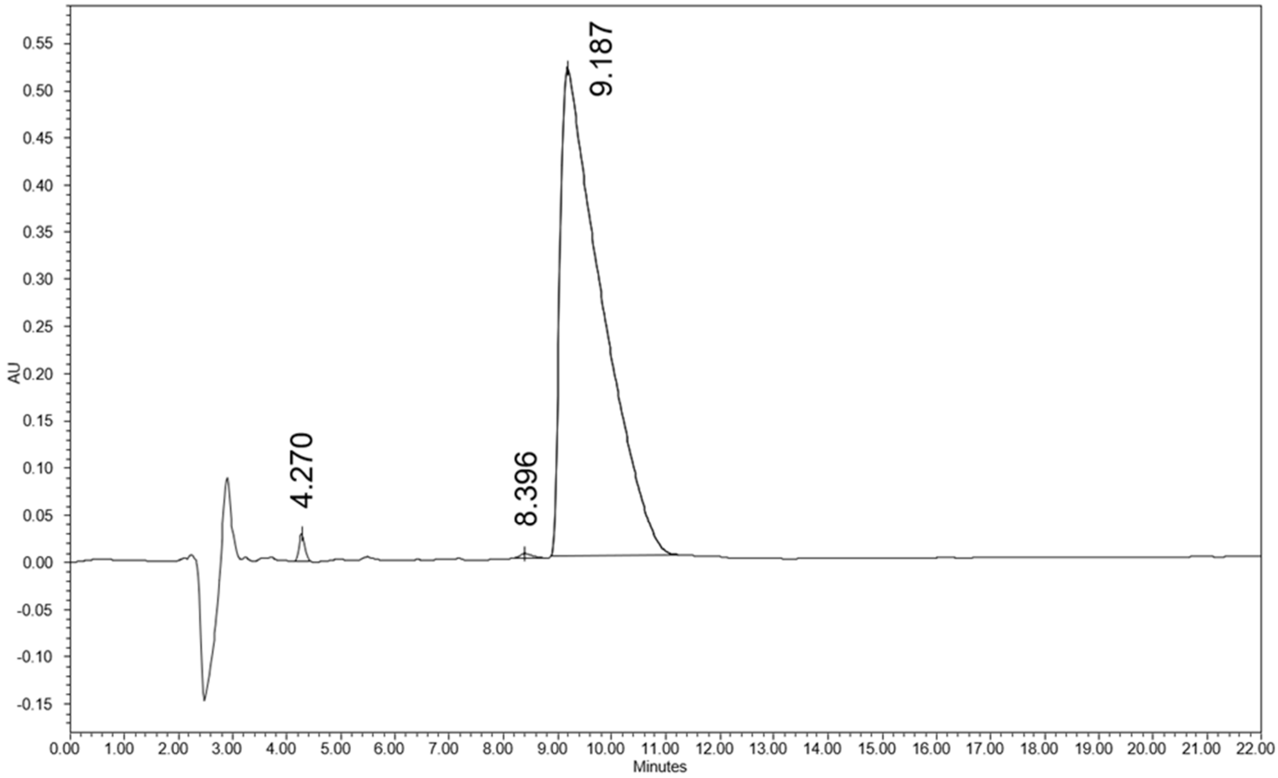

Figure S1. Analytical RP-HPLC chromatogram of purified S-X-HVP peptide. The analysis conditions were: Nova-Pak HR C_{18} (4 µm, 60 Å, 3.9 × 300 mm, Waters), injection volume, 35 µL of 1 mg/mL peptide solution; flow rate, 1 mL/min; eluent A, 0.05% TFA in water; eluent B, 0.05% TFA in CH_3CN; gradient, from 13%B to 23%B in 20 min, detection at 214 nm. The retention time results 9.187 min.

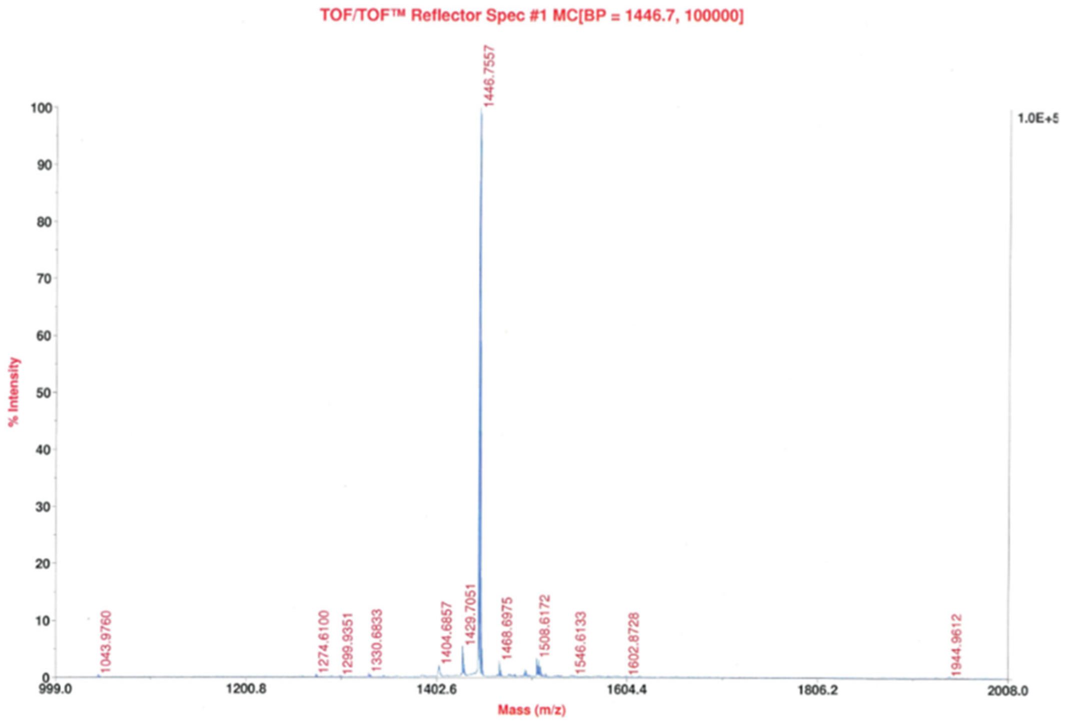

Figure S2. Mass analysis of S-X-HVP peptide. MALDI-TOF evaluation confirmed the identity of the purified peptide. S-X-HVP: experimental mass = 1446.7 Da, theoretical mass = 1446.6 Da.

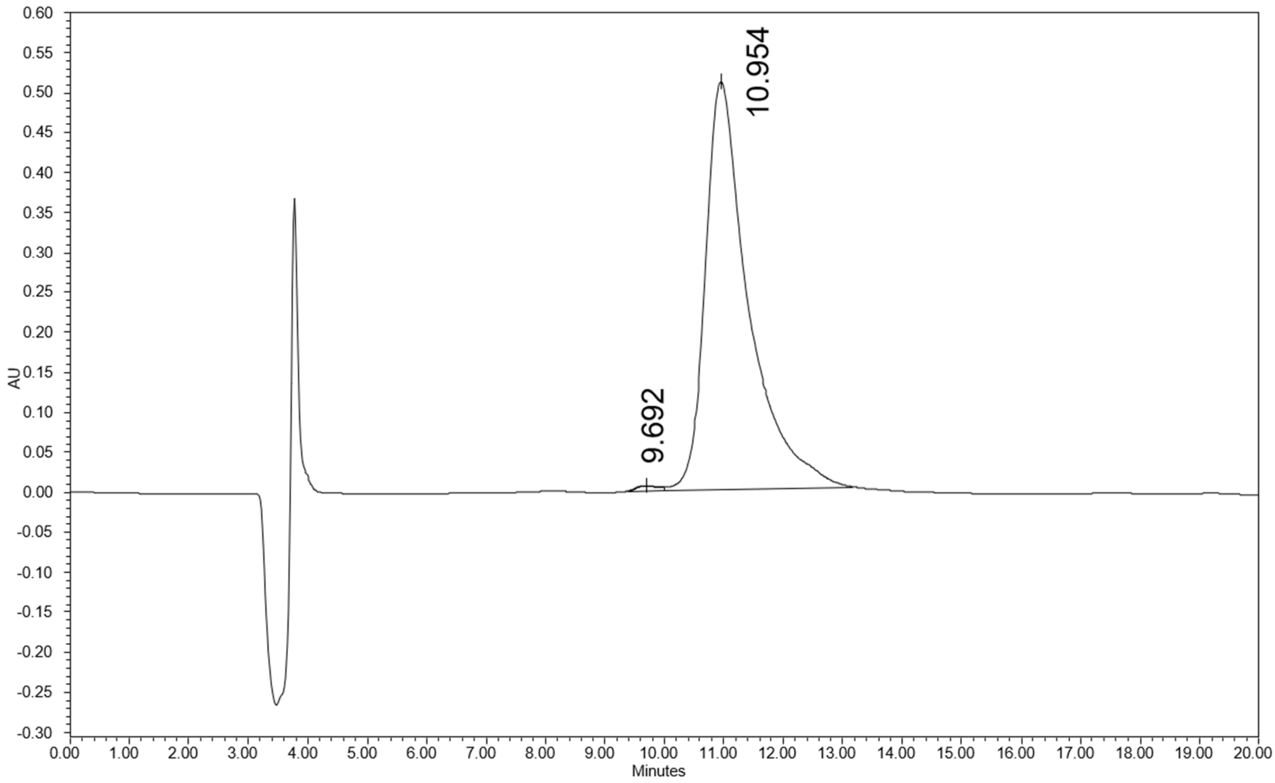

Figure S3. Analytical RP-HPLC chromatogram of purified GBMP1a. The analysis conditions were: Jupiter C_{18} (5 µm, 300 Å, 4.6 × 250 mm, Phenomenex), injection volume, 100 µL of 1 mg/mL peptide solution; flow rate, 1 mL/min; eluent A, 0.05% TFA in water; eluent B, 0.05% TFA in CH_3CN; gradient, from 31%B to 41%B in 20 min, detection at 214 nm. The retention time results 10.954 min.

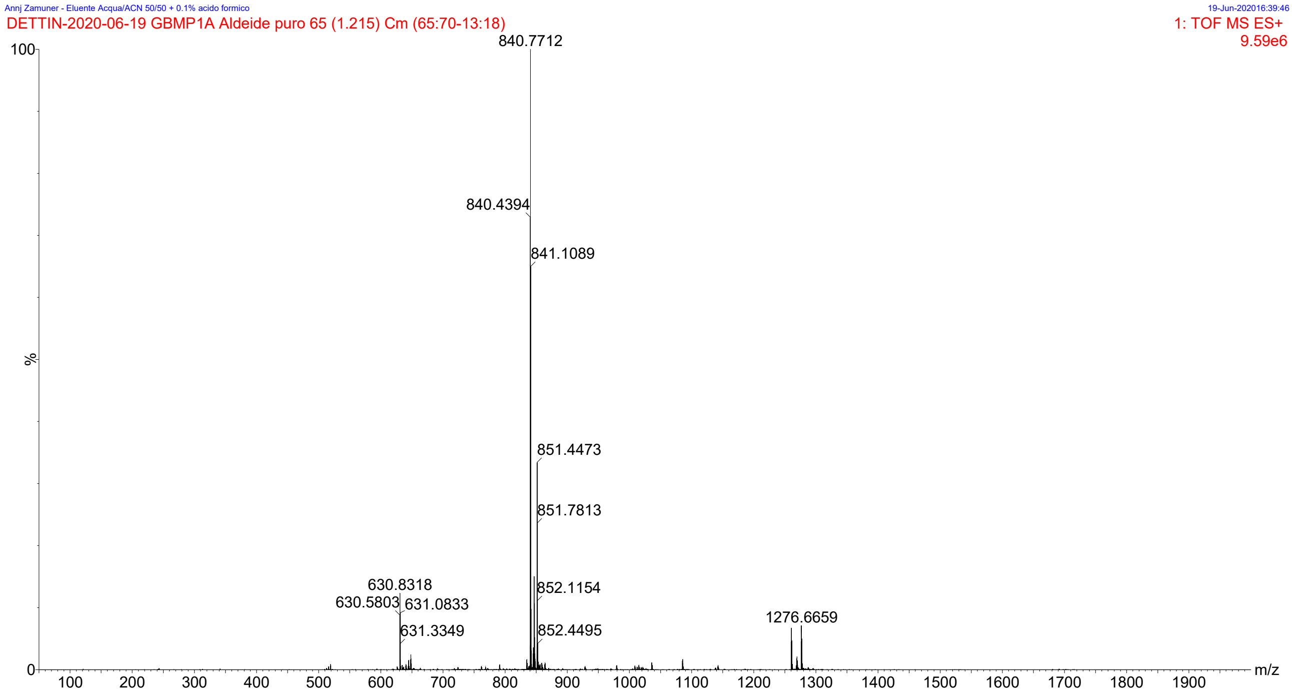

Figure S4. Mass analysis of GBMP1a peptide. ESI-TOF evaluation confirmed the identity of the purified peptide. GBMP1a: experimental mass deconvolution = 2519.3 Da, theoretical mass = 2519.8 Da.

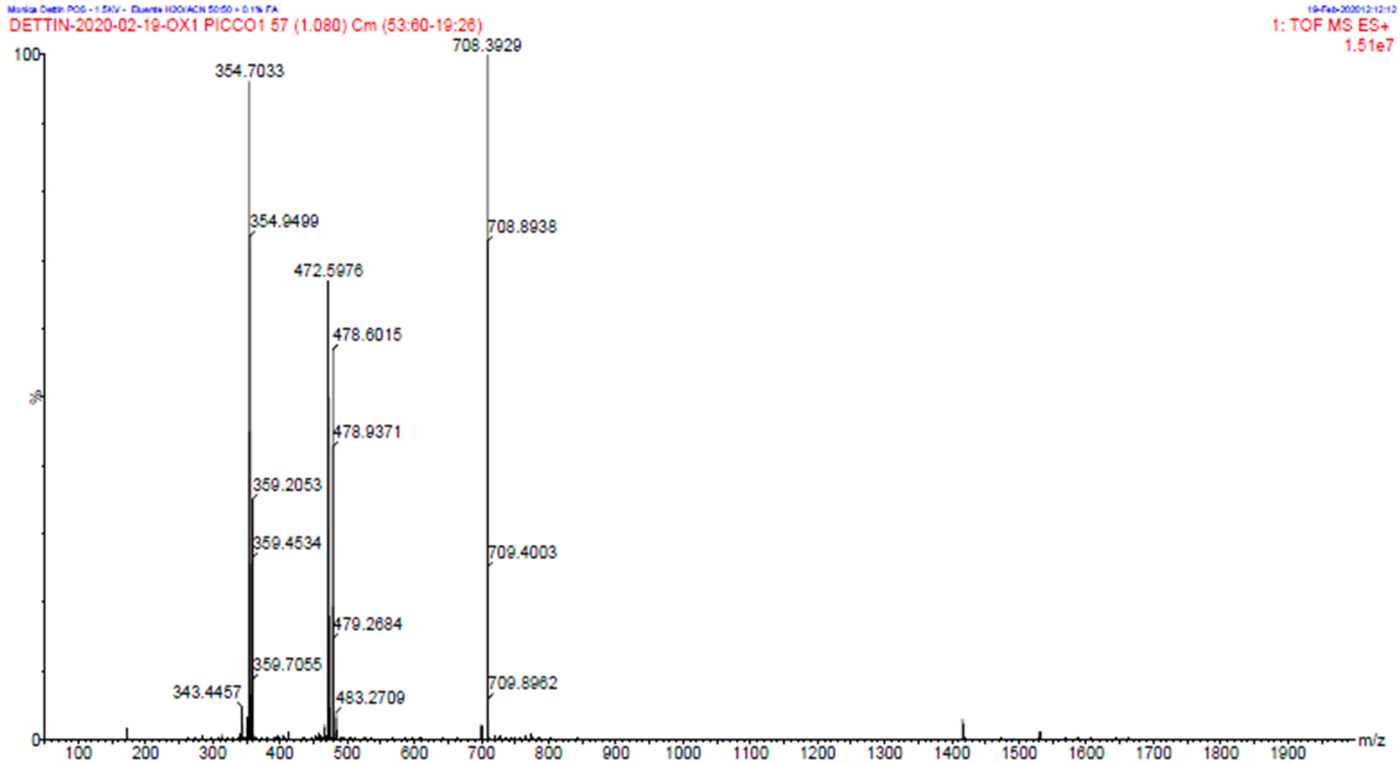

Figure S5. Mass analysis of ald-X-HVP peptide. ESI-TOF evaluation confirmed the identity of the purified peptide. Ald-X-HVP: experimental mass = 1414.8 Da, theoretical mass = 1415.6 Da.
